# Biomolecular strategy for designing antibiotic–silver nanoparticles conjugate via nitrate reductase mediated β-lactamase inhibition with molecular docking insights

**DOI:** 10.1038/s41598-025-30539-8

**Published:** 2026-01-03

**Authors:** Gerges Gad Faheem, Bahig A. El Deeb, Mohamed Ismeal, Mahmoud S. Bakhit

**Affiliations:** 1 Department of Botany and Microbiology, Faculty of Science, Sohag University, Sohag, 82524 Egypt; 2 Department of Chemistry, Faculty of Science, Sohag University, Sohag, 82524 Egypt

**Keywords:** Biosynthesis mechanism, Amp–AgNPs characterizations, In silico docking, Iodometric assay, Β-lactamase inhibition, Drug delivery, Biochemistry, Biotechnology, Drug discovery, Microbiology

## Abstract

In this study, mechanistic analysis using SDS-PAGE identified a 58 kDa protein as the biomolecule responsible for AgNPs biosynthesis and capping. Colorimetric microplate-based assay confirmed the protein as nitrate reductase, with structural preservation evidenced by a 29.68% activity increase (1.856 to 2.407 U/g) following AgNPs synthesis. Functionalization of AgNPs with ampicillin was indicated by SPR shift from 422.5 to 340.5 nm and disappearance of the FTIR band at 1736 cm⁻¹. Amp–AgNPs conjugate was stable (3 months), semi-spherical crystalline, polydisperse (PDI: 0.192), average diameter of 27.26 nm, zeta potential of − 24.9 mV, and showed broad pH (1–9) and thermal (5–55 °C) stability. Docking analysis revealed strong binding of ampicillin within the nitrate reductase catalytic pocket through hydrogen bonding, hydrophobic, and electrostatic interactions, confirming the conjugate stability. Amp–AgNPs (50 µg/mL) exhibited potent antibacterial activity against β-lactamase-producing bacteria with inhibition zones of 27.3 mm (*Escherichia coli*), 25.0 mm (*Enterococcus faecalis*), and 26.3 mm (*Staphylococcus aureus*), and MICs of 3.3, 4.7, and 4.3 µg/mL, respectively. SEM analysis revealed severe structural changes, indicating synergistic membrane disruption and antibiotic delivery. Amp–AgNPs potently inhibited β-lactamase in the iodometric assay, supporting their potential as alternative therapeutic agents. Future studies should focus on in vivo efficacy and expand this strategy to additional drug delivery applications.

**Supplementary Information:**

The online version contains supplementary material available at 10.1038/s41598-025-30539-8.

## Introduction

Nanoparticles have become transformative tools in medicine, biotechnology, and environmental applications due to their unique physicochemical properties at the nanoscale^1^. Their synthesis can be achieved through physical, chemical, or green methods categorized into top-down and bottom-up approaches based on the formation mechanism^2^. Top-down methods involve breaking down bulk materials into nano-scale particles using mechanical or thermal forces, producing uniform nanoparticles but requiring complex equipment, additional energy inputs, and lacking stabilizers to prevent agglomeration^3,4^. The bottom-up approach assembles nanoparticles from molecular precursors through nucleation and growth, typically using chemical or biological synthesis pathways involving metal salts^5^. While chemical synthesis allows for rapid production, it may involve hazardous substances, which can restrict medical applications^6^. In contrast, biological synthesis offers an environmentally friendly alternative, utilizing molecules derived from plants and microorganisms^7,8^. Fungi, particularly endophytic fungi, are efficient biological agents for nanoparticle synthesis due to their ease of cultivation and high secretion of enzymes and proteins that enhance nanoparticle stability^9,10^. Endophytes produce diverse bioactive compounds and extracellular enzymes with industrial and therapeutic relevance^11–13^. Among these, nitrate reductase (NR) is an enzyme implicated in the extracellular synthesis of AgNPs, facilitating the reduction of metal ions^14–16^.

Nitrate reductase, a molybdo-flavoprotein, catalyzes the nitrate reduction to nitrite using pyridine nucleotides as electron donors^17^. Nitrate reductase is categorized based on its coenzyme specificity: NADH-specific in higher plants, NADH/NADPH in algae, and NADPH-specific in fungi^18,19^. It is a multidomain enzyme comprising the prosthetic groups molybdopterin, Fe-heme, and FAD (flavin adenine dinucleotide) in a 1:1:1 stoichiometry that mediates an electron transfer from NAD(P)H to nitrate^20^. The biosynthesis of AgNPs is proposed to involve NADH as an electron donor and NADH-dependent nitrate reductase as a catalytic mediator in the reduction of silver ions to metallic silver, a function consistent with the enzyme’s electron transfer capabilities and structural composition^21,22^.

Capping agents are molecules that encase and stabilize AgNPs, preventing agglomeration, and ensuring their stability^23^. Proteins function effectively as green capping agents in the synthesis of nanoparticles due to their capacity to bind to metal surfaces through functional groups like amine, carboxyl, and thiol residues^24^. These biomolecules not only stabilize AgNPs by curtailing agglomeration but also play a crucial role in influencing their surface charge (zeta potential), size, and shape^25^. Protein-capped nanoparticles enhance biocompatibility, offering a biodegradable, non-toxic alternative to synthetic stabilizers, and enable surface modifications for various medical applications^26^. The stabilization of nanoparticles by capping agents is mediated through various mechanisms, including steric hindrance, depletion stabilization, electrostatic interactions, hydration forces, and van der Waals forces^27^. Nitrate reductase primarily facilitates the bio-reduction of silver ions while associated proteins possibly including the enzyme itself may adsorb onto the nanoparticle surface functioning as capping agents that contribute to their stabilization^28,29^.

Functionalization of AgNPs involves the modification of the nanoparticle surface with specific ligands, functional groups, or biomolecules to improve physicochemical properties and biological interactions^30^. This strategy improves stability, enables targeted delivery, and facilitates controlled drug release^31^. The synthesis of AgNPs functionalized with antibiotics holds potential medical applications, making the elucidation of the binding mechanisms on their surface crucial for further study^32^. By pairing specialized properties of metallic nanoparticles with antibiotics, this method offers a dynamic approach to counteracting the growing threat of bacterial resistance^33^. Green-synthesized metallic nanoparticles, when combined with antibiotics, can produce synergistic effects that enhance antibacterial activity, disrupt biofilms, and reduce the likelihood of resistance development^34^. Conjugates of antibiotics and AgNPs may be considered an alternative treatment to resistant bacterial strains because combined formulations of antibiotics-nanoparticles not only reduce the dose of medicine but also minimize the chances of toxicity^35^. Multidrug-resistant microorganisms are being targeted through the functionalized nanoparticles with less potent antibiotics to enhance their antimicrobial efficacy^36^.

The global rise of multidrug-resistant (MDR) bacteria poses a serious public health threat, largely due to their production of β-lactamases. These enzymes hydrolyze the β-lactam ring and inactivate widely used β-lactam antibiotics such as penicillin, cephalosporins, monobactams, and carbapenems^37,38^. These enzymes are characteristic of MDR strains, which severely reduce the efficacy of β-lactam antibiotics, including penicillin, ampicillin, and cephalosporin^39^. β-lactam antibiotics act by inhibiting penicillin-binding proteins, preventing peptidoglycan cross-linking, and ultimately causing bacterial cell lysis. To counteract resistance, β-lactam antibiotics are commonly combined with β-lactamase inhibitors such as clavulanic acid, sulbactam, and tazobactam. These inhibitors protect the antibiotic by binding to the enzyme, but their activity is limited against certain β-lactamase types, particularly metallo-β-lactamases^40^. This growing challenge highlights the urgent need for novel approaches, including antibiotic–nanoparticle conjugates, to enhance antibacterial efficacy and overcome β-lactamase-mediated resistance^41^.

Ampicillin is a broad-spectrum β-lactam antibiotic that belongs to the aminopenicillin subclass. It is effective against a variety of Gram-positive and some Gram-negative bacteria^42^. Its clinical effectiveness has been significantly reduced due to widespread resistance, primarily mediated by β-lactamase enzymes^43^. To counteract this, ampicillin is often administered in combination with sulbactam, a β-lactamase inhibitor^44^. However, the effectiveness of such combinations remains limited, as many β-lactamase variants, particularly extended-spectrum and metallo-β-lactamases, are not inhibited by sulbactam^45^.

In silico molecular docking has emerged as a pivotal tool in fundamental and applied biological research, enabling the rational investigation of molecular interactions^46^. These computational studies facilitate the elucidation of interaction mechanisms between functionalized nanoparticles and biomolecular targets, providing structural and energetic insights that complement experimental observations^47^. Moreover, in silico methods provide powerful, cost-effective alternatives in drug design by enabling rapid predictions of physicochemical properties without requiring compound isolation, though experimental validation remains essential^48^. Such approaches can accelerate the identification and design of antibacterial compounds capable of targeting MDR strains with diverse resistance mechanisms^49^. Advanced bioinformatics platforms allow for the prediction of binding affinities, identification of active site preferences, and assessment of structural compatibility among AgNPs capping proteins and antibiotics, thereby uncovering potential modes of action and synergistic effects^50^. Molecular docking specifically forecasts ligand binding orientations and affinities to target proteins by evaluating ligand shapes and optimal binding conformations^51^. Importantly, integrating molecular modelling with experimental validation not only increases predictive accuracy but also bridges the gap between computational hypotheses and clinically relevant outcomes^52^. Computational approaches provide in-depth insights into nanoparticle behavior and interactions within complex biological systems^53^.

This study presents an integrated experimental and computational approach to investigate the role of fungal nitrate reductase in the green synthesis, capping, and functionalization of AgNPs produced by *Talaromyces funiculosus* (SUMCC 22011). The novelty of this research lies in recognizing nitrate reductase not only as a biocatalyst for the biosynthesis of AgNPs but also as a capping agent. Ampicillin (Amp) was conjugated with the biosynthesized AgNPs in a defined stoichiometric ratio, resulting in a stable nano-antibiotic conjugate (Amp–AgNPs). Comprehensive characterization techniques, including ultraviolet–visible spectroscopy (UV–Vis), X-ray diffraction (XRD) analysis, dynamic light scattering (DLS), zeta potential analysis, Fourier transform infrared spectroscopy (FTIR), scanning electron microscopy (SEM), and high-resolution transmission electron microscopy (HR-TEM), confirmed the successful formation and structural integrity of ampicillin–AgNPs conjugate (Amp–AgNPs). The physicochemical stability of Amp–AgNPs was assessed under varying pH and temperature conditions. Molecular docking was utilized to model the active site architecture of nitrate reductase and its interactions with ampicillin, providing mechanistic insights into the formation of Amp–AgNPs conjugate. The antibacterial activity of Amp–AgNPs was assessed, and the associated morphological alterations in bacterial cells were confirmed by SEM. The β-lactamase inhibition ability of Amp–AgNPs was evaluated using the iodometric assay. This integrative experimental and computational approach provides a sustainable and innovative platform for AgNPs biosynthesis and functionalization for biomedical applications.

## Results and discussion

### Functional verification of nitrate reductase in the biosynthesis and capping of AgNPs

**Fig. 1:**
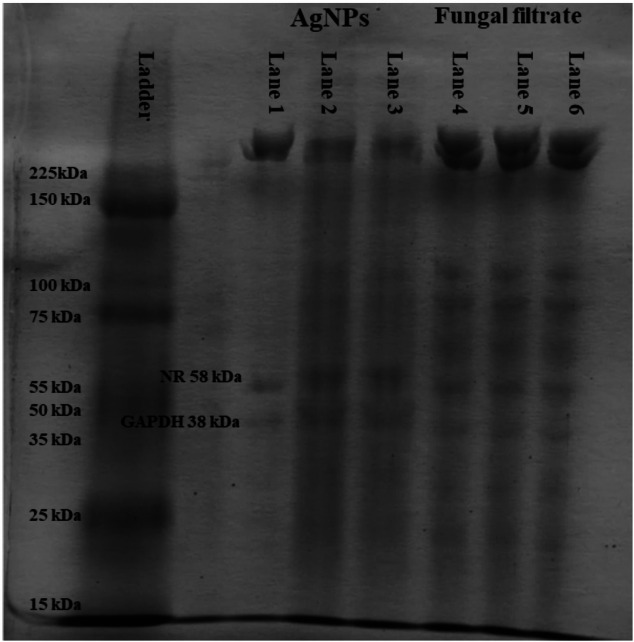
SDS-PAGE analysis for detecting proteins involved in the biosynthesis and capping of AgNPs: (**Ladder**) Protein molecular weight marker, (**Lanes 1–3**) Protein capped AgNPs, (**Lanes 4–6**) Proteins from *T. funiculosus* filtrate, (**GAPDH**) Internal protein reference (38 kDa), (**NR**) Nitrate reductase band at 58 kDa.

*Talaromyces funiculosus* (SUMCC 22011) is an endophytic fungus isolated from the wild medicinal plant *Euphorbia hirta* and identified based on morphological characteristics and phylogenetic analysis, as reported in our previous study El deeb et al.^8^. Sodium dodecyl sulfate-polyacrylamide gel electrophoresis (SDS-PAGE) analysis of *T. funiculosus* filtrates and the corresponding biosynthesized AgNPs revealed distinct protein profiles (Fig. 1). The complete uncropped gel image is provided in the supplementary information (see Supplementary Fig. S1 online). Fungal filtrates showed protein bands at 58, 75, and 100 kDa. A prominent band consistently observed at 58 kDa across all fungal filtrates and AgNPs derived from three independent fungal patches indicates the presence of a protein involved in the synthesis and capping of AgNPs. A colorimetric microplate-based assay for NR activity was performed to confirm the identity of the observed protein band. The results validated the enzymatic activity associated with nitrate reduction in the fungal filtrate and the AgNPs-capped protein. The NR activity of the fungal filtrates was 1.856 ± 0.063 U/g biomass, and the activity increased to 2.407 ± 0.077 U/g for the AgNPs, indicating 29.68% enhancement following biosynthesis. The detection of enzymatic activity at the same molecular weight position in the SDS-PAGE further supports the presence of functionally active NR in both fractions. The results confirm that NR activity is preserved during AgNPs biosynthesis and remains catalytically active when associated with the nanoparticle surface.

Several studies have highlighted the involvement of proteins in AgNPs biosynthesis. For instance, *Macrophomina phaseolina* exhibited an 85 kDa protein band, identified as a stabilizing agent for AgNPs^54^. Similarly, extracellular proteins with molecular masses of 45 kDa were reported in *Aspergillus niger* cell filtrates^28^, while *Aspergillus flavus* secreted proteins of 32 and 35 kDa, in the culture filtrate and bound to AgNPs^55^. *Trichoderma asperellum* was shown to produce protein bands at 70 and 55 kDa, which were directly linked to nitrate reductase activity and AgNPs biosynthesis^56^.

For further confirmation that the 58 kDa band belongs to NR, A colorimetric assay was employed for the fungal filtrates and the AgNPs after drying at room temperature and suspended in ionized distilled water. To the best of our knowledge, this is the first quantitative study to report a significant enhancement in NR activity following the biosynthesis of AgNPs in a fungal system. Nitrate reductase is an enzyme produced by various microorganisms, plays a crucial role in the extracellular biosynthesis of AgNPs by catalyzing the reduction of silver ions to elemental silver via an NADH-dependent electron transfer mechanism^57^. While it typically facilitates the conversion of nitrate to nitrite, the capacity of the enzyme to mediate electron transfer is effectively utilized in AgNPs biosynthesis to reduce silver ions, leading to their nucleation and subsequent formation^58^. This mechanism is conserved across a diverse range of microbial taxa including fungi, yeasts, and bacteria^14,59,60^. Among filamentous fungi, *Fusarium oxysporum* has been extensively characterized for its NR-mediated nanoparticle synthesis, with reported enzymatic rates up to 220 nmol/h/mL^61^. Similarly, *Aspergillus oryzae* and *Trichoderma reesei* have been shown to utilize nitrate reductase effectively in AgNPs biosynthesis^15,62^. Intra-genus variability in NR activity has also been documented among *Aspergillus* species, with *A. fumigatus* exhibiting the highest activity, followed by *A. clavatus* and *A. niger*, while *A. flavus* demonstrated comparatively lower enzymatic performance^63^. Other fungal genera have also displayed considerable NR activity. For instance, *Penicillium* spp. exhibited significant enzymatic activity quantified at 270 nmol/h/mL^64^. Among yeasts, *Cryptococcus laurentii* and *Rhodotorula glutinis* showed NR activities of 266.56 nmol/h/mL and 216.85 nmol/h/mL, respectively^60^. In bacterial systems, *Bacillus subtilis* demonstrated nitrate reductase activity of 152 nmol/h/mL^59^, while crude metabolite extracts from *Escherichia coli* displayed activity levels equivalent to 2.18 U/mL^65^. The significant increase in nitrate reductase activity in the AgNPs-associated fraction of *T. funiculosus* highlights a functional integration between the enzyme and the nanoparticle matrix. This interaction enhances catalytic efficiency, suggesting a stable nano–bio configuration with potential applications in developing high-performance bio-catalytic systems for therapeutic and industrial use.

### Physicochemical and morphological validation of Amp–AgNPs formation

**Fig. 2:**
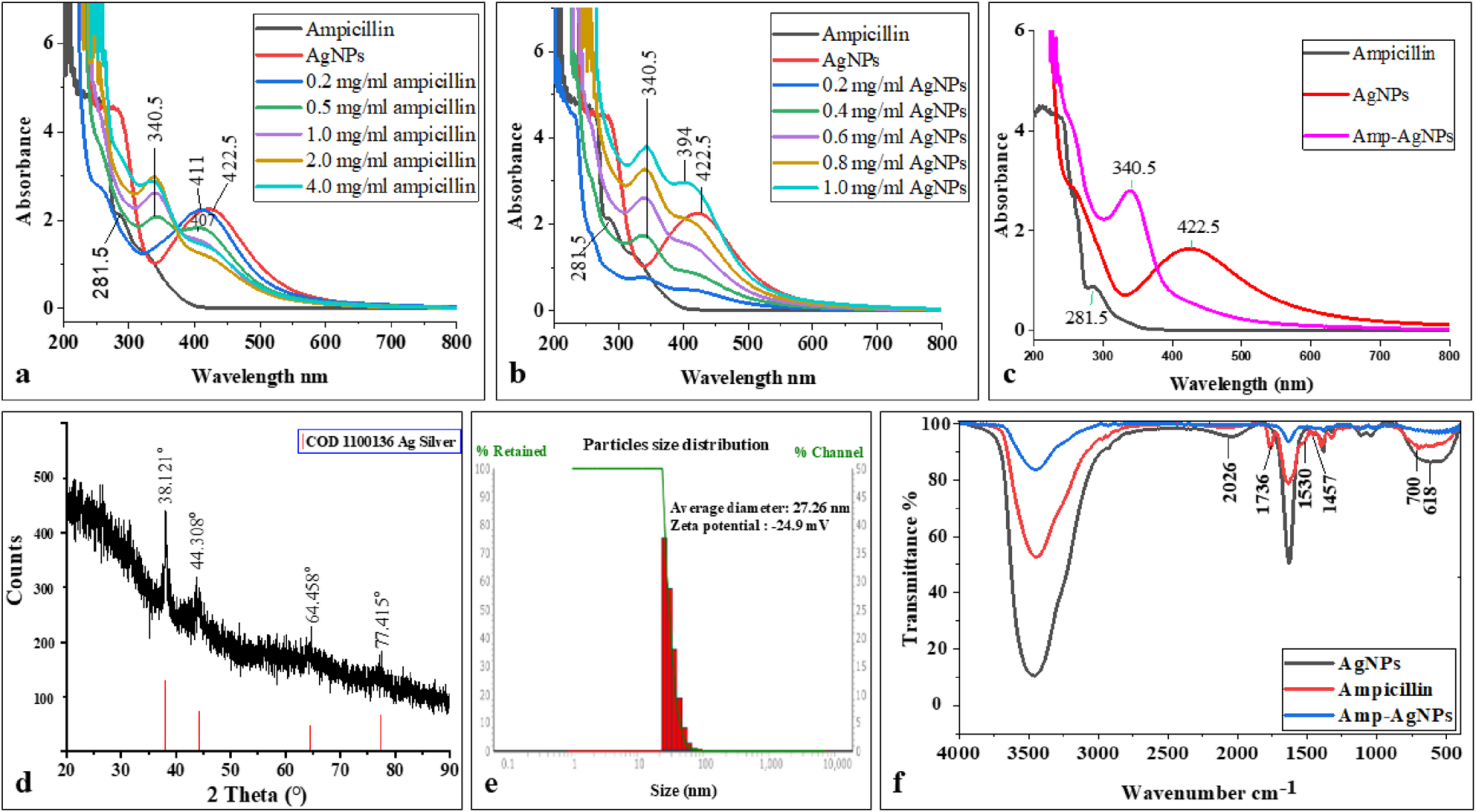
Characterization of Amp–AgNPs: (**a**–**c**) UV-visible absorption spectra of Amp–AgNPs synthesized using varying concentrations of ampicillin, using varying concentrations of AgNPs, and at optimal concentration of ampicillin and AgNPs, respectively, (**d**) XRD pattern, (**e**) DLS analysis, (**f**) FTIR spectra of ampicillin, AgNPs, and Amp–AgNPs.

UV–visible spectroscopy provided initial confirmation of the successful formation of Amp–AgNPs conjugate. As shown in Fig. 2a, increasing the ampicillin concentration (0.2–4.0 mg/mL) resulted in a gradual shift of the characteristic AgNPs SPR peak at 422.5 nm, along with the appearance of a secondary peak at 340.5 nm. These spectral changes indicate strong interactions between the β-lactam antibiotic and the nanoparticle surface. Similarly, varying the AgNPs concentration from 0.2 to 1.0 mg/mL (Fig. 2b) led to enhanced SPR intensity and peak shifts, supporting the concentration-dependent modulation of nanoparticle optical properties. As shown in Fig. 2c, under the optimized synthesis conditions (1.0 mg/mL AgNPs and 1.0 mg/mL ampicillin), the Amp–AgNPs conjugate exhibited a distinct SPR peak at 340.5 nm. This characteristic peak confirms the formation of the novel hybrid nanostructure. The conjugate remained stable over 3 months at room temperature, as evidenced by unchanged UV–visible spectra, indicating preserved structural integrity and colloidal stability throughout the storage period.

Functionalization of AgNPs with biomolecules enhances their stability and provides properties such as target specificity, fluorescence, and antimicrobial activity, supporting their broad applicability in biomedical nanotechnology^30^. The AgNPs used for ampicillin functionalization were biosynthesized by *T. funiculosus* and characterized by our previously study El deeb et al.^8^ as spherical crystalline, stable (6 months), and mono-dispersed (PDI: 0.007), exhibiting SPR at 422.5 nm, average diameter of 34.32 nm, and zeta potential of -18.41 mV. The pronounced 82 nm shift in Amp–AgNPs UV–visible spectra reflect significant change in the electronic environment and surface characteristics of the nanoparticles, supporting successful functionalization. Such molecular interactions promoted charge transfer and altered the electronic structure, particularly through transitions from non-bonding (n) orbitals to antibonding pi (π*) orbitals, ultimately leading to a blue shift in the SPR band, as described in the study by Onyangore et al.^66^. The noteworthy 82 nm shift sharply contrasts with the more minor shifts observed in previous studies, such as the shift from 408 nm to 427 nm upon ampicillin functionalization^67^ and a minor shift from 436 nm to 442 nm upon AgNPs functionalized with ampicillin^68^. In addition, minimal shifts from 438 nm to the range of 421–426 nm were noted with cephalosporin conjugates^69^, and the shift from 438 nm to 477 nm occurring after conjugation with ciprofloxacin^70^, while amoxicillin functionalization resulted in a distinct SPR shift from 425 nm to 475 nm^71^. Other reports have shown SPR bands for silver-drug conjugates generally remaining within the 400–450 nm range^72^, while ampicillin-functionalized AgNPs displayed an SPR at 390 nm^73^. In the direct synthesis of AgNPs using ampicillin as a reducing and stabilizing agent, Khatoon et al.^74^ reported an SPR peak at 406 nm, confirming ampicillin capability in nanoparticle synthesis; however, the peak remained within the expected range for AgNPs. The consistent SPR band at 340.5 nm for 3 months further confirms the long-term stability of the Amp–AgNPs, demonstrating their suitability for potential therapeutic applications. Similar stability results were reported by Brown et al.^75^.

The XRD pattern of the Amp–AgNPs conjugate (Fig. 2d) displayed characteristic diffraction peaks at 2θ values around 38.121°, 44.308°, 64.458°, and 77.415°, corresponding to the (111), (200), (220), and (311) planes of face-centered cubic (FCC) silver (Pattern: COD 1100136 Ag Silver). This pattern was consistent with the XRD of AgNPs reported in our previous study by El deeb et al.^8^, confirming that conjugation did not alter the crystalline structure of AgNPs. The presence and retention of these peaks after conjugation demonstrated that the metallic silver core remained intact despite ampicillin functionalization. The conjugate also maintained its phase purity, confirming that no structural alterations occurred during functionalization. Preserving both crystallinity and phase purity is crucial for ensuring nanoparticle stability and enhancing biological performance. Such non-alteration of the silver crystalline core upon functionalization has been consistently reported in earlier studies, indicating that conjugation occurs mainly at the surface without affecting the metallic lattice^68,71,76^.

DLS analysis of synthesized Amp–AgNPs displayed a size range of 22.2 to 39.46 nm, with an average diameter of 27.26 nm, while zeta potential measurements indicated a negative surface charge of − 24.9 mV (Fig. 2e). The reduction in hydrodynamic size from 34.32 (AgNPs) to 27.26 nm following ampicillin conjugation is attributed to the active functional groups of ampicillin, which interact with and reorganize the nanoparticle surface, increase surface charge, and compress the stabilizing sheath. These modifications result in a smaller and more stable particle size, as evidenced by the increase in negative zeta potential from − 18.6 to − 24.9 mV. Similar findings were reported by Lopez-Carrizales et al.^77^ where the conjugation of ampicillin led to a reduction in AgNPs size from 8.57 to 4.01 nm. This decrease was attributed to ampicillin favoring the homogeneous dispersion of the nanoparticles and enhancing colloidal stability, as reflected by an increase in negative zeta potential from − 40.01 to − 51.00 mV. Comparable effects have also been observed with tetracycline, where surface modification influences nanoparticle dispersion, surface charge, and overall colloidal stability^78^. In a study by Rogowska et al.^32^ functionalization of AgNPs with ampicillin resulted in an increase in particle size and a shift in zeta potential toward less negative values, indicating that the impact on colloidal stability may vary depending on the capping agents and synthesis conditions. In contrast, Adil et al.^69^ observed larger hydrodynamic diameters in antibiotic-functionalized AgNPs compared to AgNPs, attributing the increase in size to the binding of antibiotic molecules to the nanoparticle surface.

FTIR spectroscopy further validated the conjugation between AgNPs and ampicillin (Fig. 2f). In the FTIR spectrum of pure ampicillin a prominent band appeared at 1736 cm⁻¹, corresponding to the carbonyl (C = O) stretching vibration. The disappearance of this band in the Amp–AgNPs spectrum suggests that the carbonyl group of ampicillin is involved in the interaction with AgNPs. The bands observed at 1457 cm⁻¹ and 1530 cm⁻¹, assigned to β-lactam ring vibrations in pure ampicillin, were not detected in the Amp–AgNPs spectrum. The disappearance of these functional group signals supports the binding of the β-lactam group during the conjugation process. Previous studies have reported only shifts in hydroxyl and amide group regions upon antibiotic conjugation, supporting the role of these groups in stabilization and surface binding^72,79^. The FTIR spectrum of AgNPs synthesized directly using ampicillin showed a shift of the amine peak from 1605 to 1625 cm⁻¹ while the β-lactam ring and other characteristic bands remained unchanged compared to pure ampicillin^74^. Also, Murei et al.^36^ observed no new functional groups upon conjugation, suggesting that the synthetic route significantly influences the surface chemistry and degree of interaction between AgNPs and antibiotics. In contrast, the presented data reveal distinct spectral features indicative of direct bonding that enhanced conjugation efficiency.

**Fig. 3:**
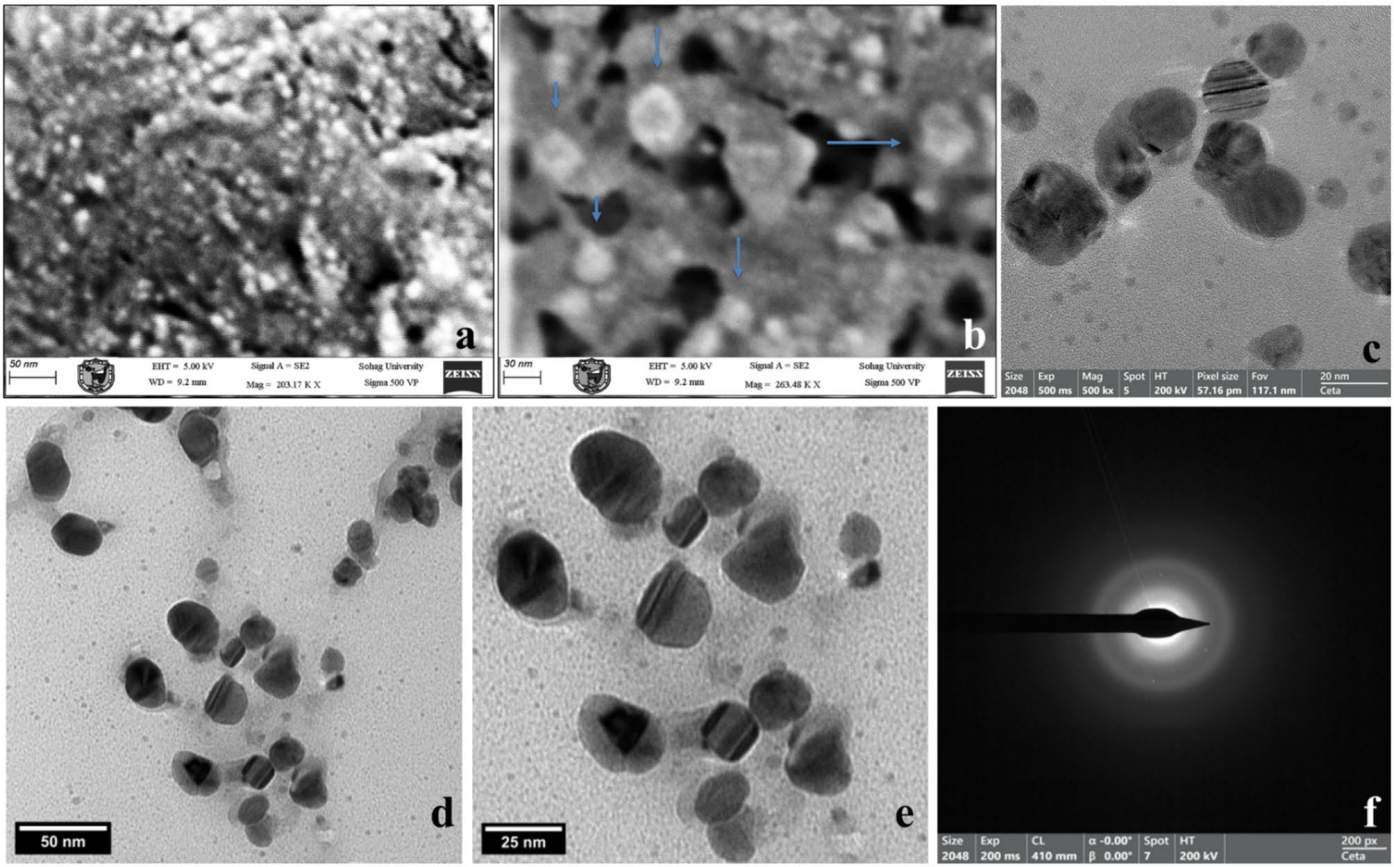
Morphological analysis of Amp–AgNPs. SEM micrographs illustrating the surface morphology of (**a**) AgNPs and (**b**) Amp–AgNPs. HR-TEM images of (**c**) AgNPs and (**d**,**e**) Amp–AgNPs showing a surrounding corona around the particles, attributed to ampicillin molecules. (**f**) SAED pattern of Amp–AgNPs.

SEM analysis of Amp–AgNPs revealed semi-spherical nanoparticles with a heterogeneous surface morphology compared to the smoother surface of AgNPs (Fig. 3a, b). This heterogeneity is attributable to functionalization-driven surface interactions.

HR-TEM imaging provided direct visual evidence of the morphology and structural organization of the Amp–AgNPs conjugate. Amp–AgNPs particles were semi-spherical, although irregular and polydisperse morphologies were also observed. A distinct surrounding corona was observed around Amp–AgNPs, confirming the presence of ampicillin molecules coating the nanoparticles (Fig. 3d, e), whereas unmodified AgNPs appeared without a corona (Fig. 3c). The calculated PDI of 0.192, obtained from TEM-based particle size distribution, indicates a relatively narrow size distribution within the low polydispersity range (0.08–0.7)^80^. As indicated by the variability in particle shape observed in TEM images, the conjugate particles are polydisperse in morphology while remaining colloidally stable, as supported by the zeta potential value of − 24.9 mV. The SAED pattern further confirmed the crystalline nature of the particles, with no detectable alterations upon conjugation with ampicillin (Fig. 3f). This combination of stability and controlled variability supports their potential suitability for biomedical applications^81^. Although the PDI increased from 0.007 for the AgNPs to 0.192 after conjugation, this rise is attributed to the surface functionalization with ampicillin.

### Stability of Amp–AgNPs under variable pH and temperature conditions

**Fig. 4:**
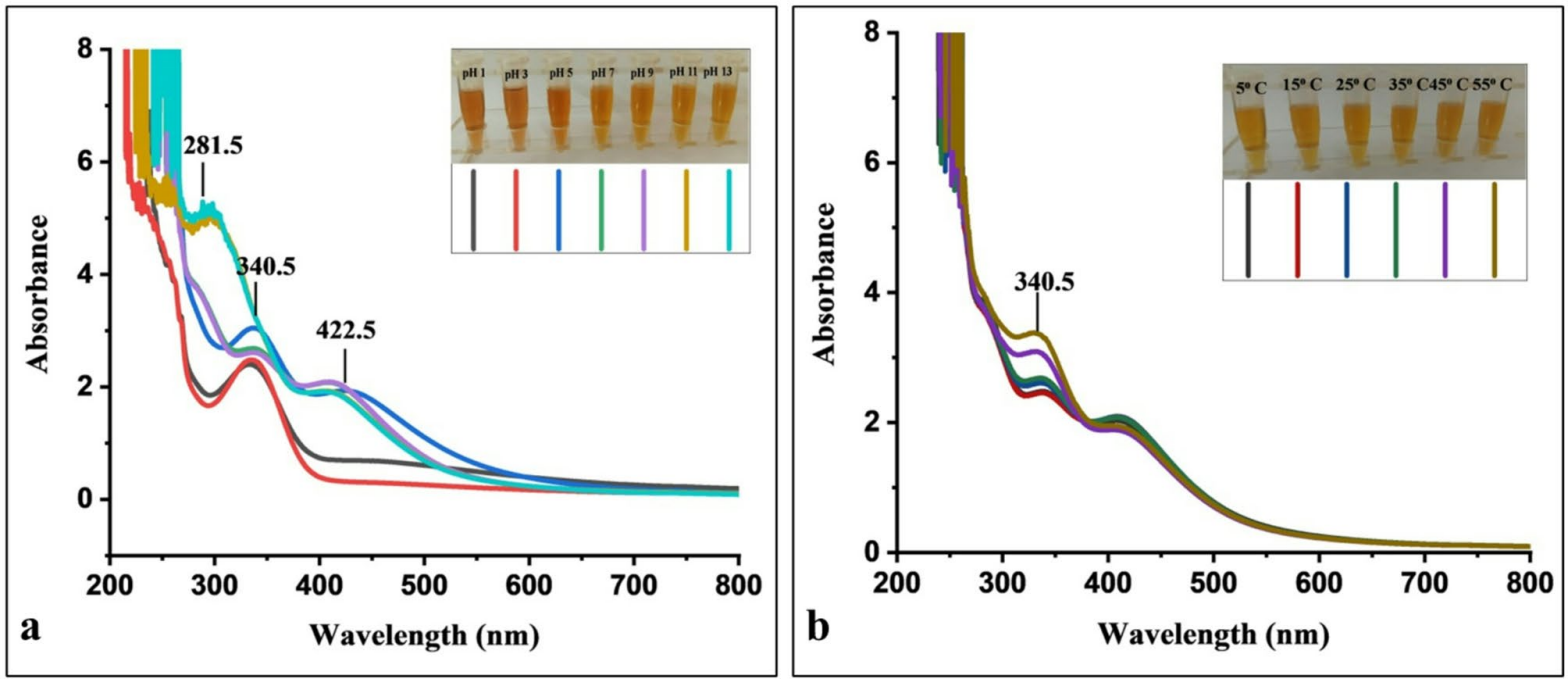
Stability of Amp–AgNPs at different: (**a**) pH values, (**b**) Temperatures.

UV–Vis spectroscopic analysis demonstrated a pH-dependent variation in the optical properties of Amp–AgNPs conjugate across a pH range of 1 to 13 (Fig. 4a). A prominent absorption peak at 340.5 nm was observed from highly acidic (pH 1) to moderately alkaline (pH 9) conditions, indicating a broad tolerance for pH variations. Elevated alkaline pH values (11–13) caused significantly diminished or poorly resolved this peak. The dissociation of the Amp–AgNPs complex at higher pH values was further supported by the appearance of distinct absorbance bands corresponding to free ampicillin (281.5 nm) and AgNPs (422.5 nm). Visual inspection revealed slightly aggregation or precipitation under strong acidic conditions after 4 h of incubation (Fig. 4a). After 48 h, the reaction mixture maintained the characteristic 340.5 nm peak, with reduced intensity, indicating time-dependent stability and some preservation of the conjugate even under extreme pH conditions. The Amp–AgNPs displayed impressive resilience across a broad pH spectrum, particularly maintaining stability at pH 5.0. This finding is consistent with research on the stability of AgNPs biosynthesized by *T. funiculosus*, which also demonstrated enhanced stability at pH 5.5^8^. Ampicillin, on the other hand, shows optimal stability at pH 7.5, experiencing over 70% degradation at pH 3.4 after 12 h, underscoring the importance of a near-neutral pH for preserving its efficacy^82,83^. Therefore, the observed stability of the conjugates at various pH levels indicates that the conjugation with AgNPs aids in maintaining the structural integrity of ampicillin, even at the extremes of its natural stability range.

The UV–vis spectroscopy analysis of the thermal stability of Amp–AgNPs was conducted across a temperature range of 5 °C to 55 °C (Fig. 4b). A consistent absorption peak at 340.5 nm was observed throughout the temperature gradient. The stability of this peak suggests that the bonding interaction between ampicillin and the AgNPs surface remains intact even at elevated temperatures. Visual inspection of the samples (inset) further supported the spectrophotometric results, showing no visible aggregation or precipitation across the temperature range (Fig. 4b). This evidence implies that Amp–AgNPs maintain colloidal stability and structural integrity up to 55 °C. Regarding thermal tolerance, biosynthesized AgNPs showed optimal stability at elevated temperatures, particularly around 60 °C^8^. Correspondingly, the Amp–AgNPs maintained their structural integrity at temperatures up to 55 °C. This stands in contrast to free ampicillin, which maintains over 90% of its initial concentration for 48 h only under refrigerated conditions (8 ± 2 °C), and for up to 24 h at 25 ± 2 °C and 30 ± 2 °C. However, at 37 ± 2 °C, its stability falls below the 90% threshold within 12 h, making it less suitable for use in warmer environments^42,84^. The enhanced thermal stability of the Amp–AgNPs indicates that nanoparticle conjugation significantly improves the physicochemical resilience of ampicillin, potentially extending its efficacy in challenging storage or treatment conditions.

Similarly, the colloidal stability of biogenic synthesized AgNPs formed stable complexes with ampicillin within a pH range of 6 to 8, with optimal stability observed at pH 7–8. Additionally, enhanced adsorption efficiency was reported at elevated incubation temperatures ranging from 4 to 42 °C^32^. The imipenem-AgNPs conjugate exhibited excellent stability, with no changes in absorbance peaks, color, visible aggregation, or clarity observed over three months of storage at 4 °C and 25 °C^85^. Also, the colloidal stability of biogenic AgNPs–nisin conjugate was confirmed at pH 4 and 8, as no shift in the SPR band or decrease in its intensity was observed, indicating that the nanoparticles remained stable and did not undergo aggregation under the tested pH conditions^86^.

### Exploring the binding mechanism of ampicillin to nitrate reductase via in Silico approaches

The 3D structure of nitrate reductase was built using homology modeling based on the obtained homologous templates. The obtained 3D structure of nitrate reductase is shown in Supplementary Fig. S2 online.

The model consists of the molybdenum cofactor (MoCo) domain that binds the MoCo where nitrate reduction occurs and this is the catalytic site center where nitrate binds and is reduced. Flavin adenine dinucleotide (FAD)-Binding domain which binds FAD for NAD(P)H-mediated electron transfer, where FAD accepts electrons from NADPH and transfers them to the MoCo center. Nitrate-binding pocket near the MoCo center, with conserved residues (e.g., Arg, His, or Ser) aiding in substrate orientation. Electron transfer pathways involving conserved cysteine residues coordinating with the MoCo centers. Potential phosphorylation sites (as seen in other fungal NRs) that regulate enzyme activity in response to nitrogen availability.

Ramachandran plot (see Supplementary Fig. S3 online) showed that almost all β-sheets, right-handed α-helices, and left-handed α-helices residues are found in the core regions (Favored Regions), with some residues in loops or flexible regions. too few residues (Gly and Pro) appear in the disallowed regions (and this is acceptable due to the large flexible loops of the model).

To find out the most active pocket, grid-based pocket analysis was performed using DOCK 6, and the most active pocket was used for docking. The detailed characteristics of all predicted pockets are summarized in Table 1, and the iso-surface representation of the selected cavity is shown in Supplementary Fig. S4 online.

**Table 1:** Predicted binding pockets of nitrate reductase and their characteristics.

Pocket	Volume (Å³)	Functional domain	Key residues	Functional role
Pocket 1	1089	Molybdenum cofactor (MoCo) binding site	His84, Arg89, Asp213, Asp221, Cys237, Trp238, Phe18, Tyr230, Phe236	Largest cavity; catalytic MoCo active site, stabilizes catalytic center, supports redox activity, positions nitrate substrate, and disulfide potential suggests redox regulation.
Pocket 2	511	FAD/NADPH-binding domain	Arg2, Lys3, Lys5, Arg124, Trp117, Tyr121, Asp122	Binds NADPH phosphate groups; aromatic residues stack with FAD’s isoalloxazine ring, and Asp122 that may hydrogen-bond to NADPH.
Pocket 3	173	Allosteric regulation site	Arg192, Glu194, Lys199, Trp203	Charged cluster suggests potential phosphorylation site; Trp203 mediates protein–protein interactions.
Pocket 4	154	Proton relay/substrate channel	Asp164, Glu165, Asp254, His163	Acidic residues (Asp164, Glu165, Asp254) facilitate proton transfer, and His163 acts as a proton shuttle during catalysis.
Pocket 5	142	Redox-sensing disulfide	Cys41, Tyr52	Potential disulfide bond formation under oxidative stress and electron transfer roles.
Pocket 6	129	Substrate access channel	Asp46, Lys47, Tyr53, Trp117	Charged (Asp46, Lys47) and aromatic (Tyr53, Trp117) residues guide nitrate into Pocket 1.
Pocket 7	122	Heme-binding interface	Arg146, Asp151, Tyr147, Tyr150	Heme propionate coordination and stabilization.
Pocket 8	118	Solvent-exposed electrostatic patch	Arg25, Lys29, Arg35	Basic residues potentially anchor nitrate reductase to membranes or interacting partner proteins.

**Fig. 5:**
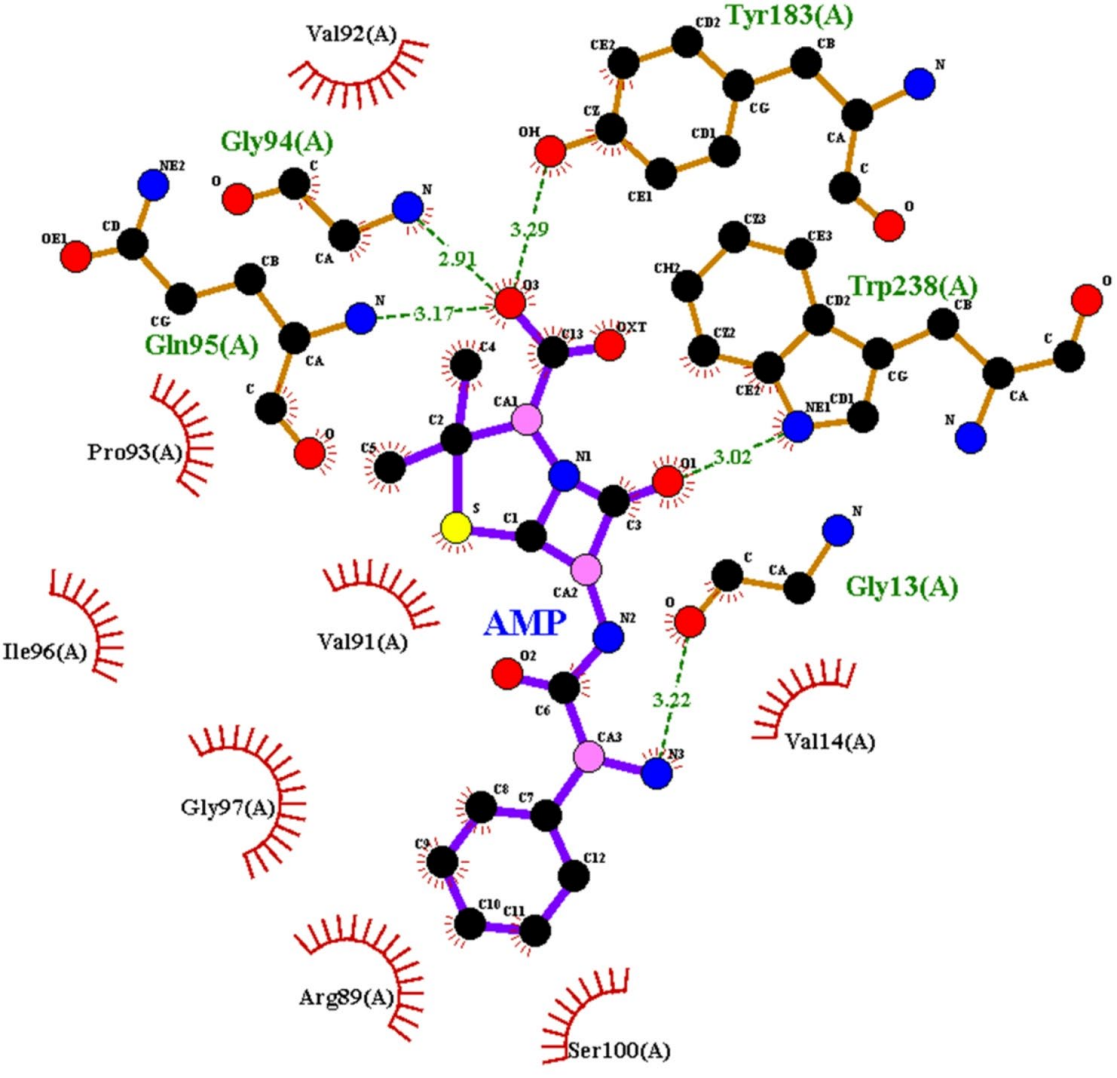
2D represents the ampicillin interaction within the active pocket of nitrate reductase enzyme. HB are shown in green line, hydrophobic and electrostatic attraction are shown in red.

The 2D structure of the lowest energy docked structure was shown in Fig. 5, where ampicillin was fitted in the active site pocket of the modeled nitrate reductase model.

It is obvious from this model that ampicillin formed HB network with residues His84, Arg89, Val92, and Gln95 where His84 and Arg89 are likely near the catalytic site (common in nitrate reductases for redox reactions). Hydrogen bonds stabilize the β-lactam ring of ampicillin, potentially interfering with substrate binding. In addition, it had electrostatic and hydrophobic interaction with residues Val14, Serl5, Thr16, Val9l, Pro93, Ser100, Leu152, Phe236, Cys237, Trp238.

This may lead to disruption of the enzyme’s charge balance near the active site. The hydrophobic cluster (Phe236, Trp238, Leu152) suggests ampicillin binds in a partially buried pocket, shielding it from solvent. The docking score was found to be -8.4 kcal/mol.

His84 & Arg89 are likely near the catalytic site, which is critical for nitrate reduction (common in NADH-dependent nitrate reductase). Ampicillin’s β-lactam ring is stabilized by these H-bonds.

Competitive or mixed-type inhibition, where ampicillin directly competes with nitrate or induces conformational changes. Arg89 (positively charged) may interact with ampicillin’s anionic carboxylate group. Ser15, Thr16 (polar) could stabilize the drug’s polar groups. Phe236, Trp238, Leu152, Val91, and Pro93 form a hydrophobic pocket, burying ampicillin’s nonpolar regions (e.g., phenyl group). Cys237 may contribute to binding via weak S/π interactions with the β-lactam ring. The hydrophobic cluster shields ampicillin from solvent, increasing residence time in the binding site. Disruption of the charge balance near the active site (due to Arg89 interaction) may impair redox chemistry.

A score of -8.4 kcal/mol suggests strong binding (typical high-affinity inhibitors range from − 7 to -12 kcal/mol).

The docking results confirmed the interaction between ampicillin and the enzyme-capped AgNPs, indicating the formation of a stable hybrid conjugate. This discovery opens new avenues for exploring the potential of drugs to conjugate with silver nanoparticles through nitrate reductase, presenting a promising strategy for improved drug delivery.

### Antibacterial activity and ultra-structural disruption of β-Lactamase-producing bacteria induced by Amp–AgNPs

**Fig. 6:**
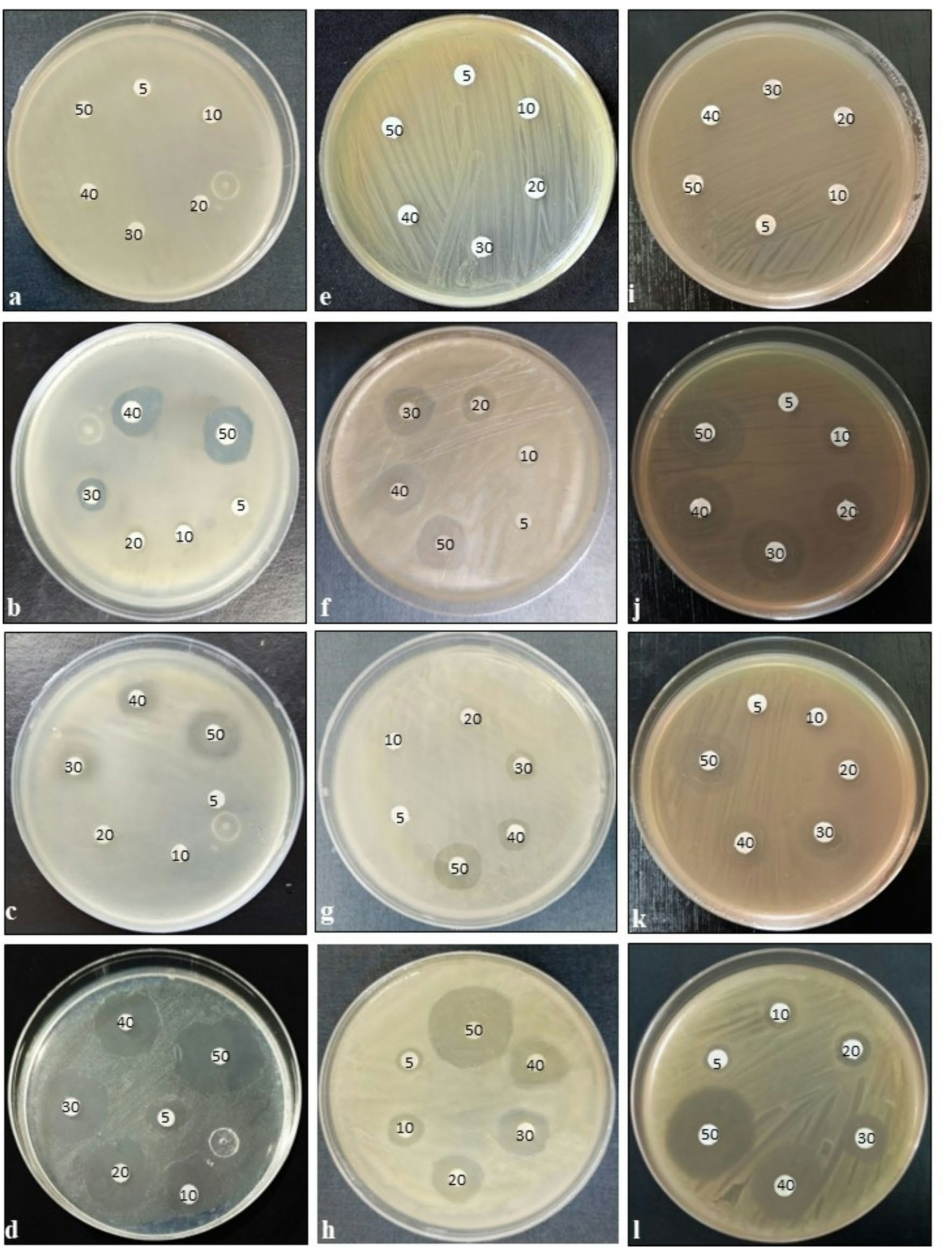
Antibacterial activities of Amp–AgNPs against β-lactamase-producing bacteria. Images show the effect on: *E. coli* (**a**–**d**), *E. faecalis* (**e**–**h**), and *S. aureus* (**i**–**l**) treated with: ampicillin (**a**, **e**, **i**), AgNPs (**b**, **f**, **j**), positive control (**c**, **g**, **k**), and Amp–AgNPs conjugate (**d**, **h**, **l**), respectively, Numerals indicate applied concentrations in µg/mL.

Amp–AgNPs demonstrated significant antibacterial activity against β-lactamase-producing bacterial strains (Table 2; Fig. 6). At low concentrations (5–10 µg/mL), Amp–AgNPs produced measurable inhibition zones ranging from 7.7 to 10.7 mm. At a concentration of 50 µg/mL, Amp–AgNPs resulted in inhibition zones measuring 27.3 ± 0.6 mm for *E. coli*, 25.0 ± 1.0 mm for *E. faecalis*, and 26.3 ± 0.6 mm for *S. aureus*. These measurements significantly exceeded those recorded for AgNPs (17.7 ± 0.6, 15.7 ± 0.6, and 16.7 ± 0.6 mm, respectively) and the positive control (ampicillin/sulbactam,15.3 ± 0.6, 13.3 ± 0.6, and 16.3 ± 0.6 mm, respectively). Ampicillin was ineffective across all tested concentrations.

**Table 2:** Inhibitory zone diameter and MIC of Amp–AgNPs against β-lactamase-producing bacteria (*p* < 0.05).

Bacterial strains		*Escherichia coli*				*Enterococcus faecalis*				*Staphylococcus aureus*			
Concentration (µg/mL)		Amp	AgNPs	Positive control	Amp–AgNPs	Amp	AgNPs	Positive control	Amp–AgNPs	Amp	AgNPs	Positive control	Amp–AgNPs
5	Inhibition zone diameter (mm)	–	–	–	8.3 ± 0.6	–	–	–	7.7 ± 0.6	–	–	–	7.7 ± 0.6
10		–	7.3 ± 0.6	–	10.7 ± 0.6	–	7.0 ± 0.0	–	11.7 ± 0.6	–	8.3 ± 0.6	7.7 ± 0.6	10.3 ± 0.6
20		–	8.3 ± 0.6	7.0 ± 0.0	14.3 ± 0.6	–	8.7 ± 0.6	7.0 ± 0.0	14.3 ± 0.6	–	12.3 ± 0.6	12.3 ± 0.6	13.7 ± 0.6
30		–	11.7 ± 0.6	9.7 ± 0.6	18.3 ± 0.6	–	12.3 ± 0.6	8.3 ± 0.6	16.3 ± 0.6	–	13.7 ± 0.6	13.3 ± 0.6	17.7 ± 0.6
40		–	14.7 ± 0.6	12.3 ± 0.6	22.7 ± 0.6	–	13.3 ± 0.6	10.3 ± 0.6	19.7 ± 1.0	–	15.7 ± 0.6	14.7 ± 0.6	21.0 ± 0.6
50		–	17.7 ± 0.6	15.3 ± 0.6	27.3 ± 0.6	–	15.3 ± 0.6	13.7 ± 0.6	25.0 ± 1.0	–	16.7 ± 0.6	16.3 ± 0.6	26.3 ± 0.6
MIC (µg/mL)		–	8.7 ± 0.6	17.3 ± 0.6	3.3 ± 0.6	–	7.7 ± 0.6	15.7 ± 0.6	4.7 ± 0.6	–	8.3 ± 0.6	9.3 ± 0.6	4.3 ± 0.6

The MIC values for Amp–AgNPs conjugate were significantly lower than AgNPs, ampicillin, and the positive control (Table 2). Amp–AgNPs MICs were 3.3 ± 0.6, 4.7 ± 0.6, and 4.3 ± 0.6 µg/mL for *E. coli*, *E. faecalis*, and *S. aureus*, respectively. However, AgNPs MIC values were 8.7 ± 0.6, 7.7 ± 0.6, and 8.3 ± 0.6 µg/mL, respectively, while for the positive control (ampicillin/sulbactam) were 17.3 ± 0.6, 15.7 ± 0.6, and 9.3 ± 0.6 µg/mL, respectively. Ampicillin demonstrated no inhibitory effect even at concentrations of 100 µg/mL. These results suggest that ampicillin conjugation with AgNPs presents a promising strategy for combating β-lactamase-producing pathogens.

**Fig. 7:**
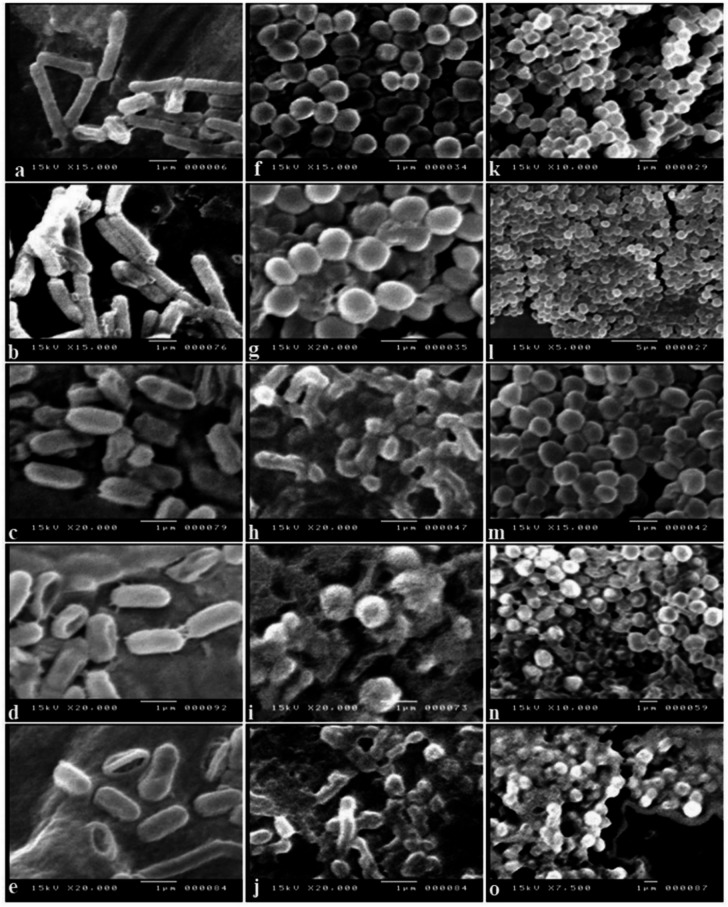
SEM analysis reveals ultra-structural disruption of β-lactamase-producing bacteria. Micrographs show morphological alterations in *E. coli* (**a**–**e**), *E. faecalis* (**f**–**j**), and *S. aureus* (**k**–**o**) subjected to: untreated cells (**a**, **f**, **k**), ampicillin (**b**, **j**, **l**), AgNPs (**c**, **h**, **m**), positive control (**d**, **i**, **n**), and Amp–AgNPs conjugate (**e**, **j**, **o**).

Scanning electron microscopy provided compelling evidence of structural damage of various β-lactamase-producing *E. coli*, *E. faecalis*, and *S. aureus* (Fig. 7). The untreated control cells displayed well-preserved morphology with smooth, intact surfaces, showing healthy cellular integrity. Cells treated with ampicillin maintained their architecture, exhibiting minimal morphological changes, which confirmed their resistance to β-lactam antibiotics. Treatment with AgNPs alone resulted in moderate membrane disruption, pore formation, and increased surface roughness. In contrast, the positive control (ampicillin/sulbactam) caused partial structural damage, including membrane wrinkling and deformation. Cells exposed to the Amp–AgNPs conjugate showed extensive morphological alteration, characterized by pore formation, membrane rupture, collapse of cellular structures, and leakage of intracellular contents. This level of damage was consistent across all tested strains. These results indicated that conjugating ampicillin with AgNPs effectively overcomes β-lactamase-mediated resistance by combining targeted membrane disruption with intracellular delivery of the antibiotic.

Amp–AgNPs synthesized in this study exhibited significantly enhanced antibacterial activity against β-lactamase-producing *E. coli*, *E. faecalis*, and *S. aureus*. This synergistic enhancement underscores the potential of AgNPs as effective carriers for conventional antibiotics, particularly in combating resistant bacterial strains. Similar results have been documented in previous studies. For instance, Alfahad et al.^87^ documented inhibition zones of 14.17 mm for Amp–AgNPs against *Salmonella typhi* using 3% aqueous AgNPs. Similarly, Rogowska et al.^32^ reported inhibition zones for Ag-CGG-Ampicillin conjugates synthesized using *Actinomycetes* CGG 11n, with zones ranging from 5.0 ± 0 mm to 12 ± 0 mm across different pathogens. Brown et al.^75^ showed that Amp–AgNPs achieved complete eradication of resistant *E. coli* and *Pseudomonas aeruginosa* within 4–6 h, compared to 6–8 h for AgNPs alone, highlighting the conjugate’s enhanced bactericidal kinetics and its potential to prevent biofilm formation. Ibraheem et al.^70^ reported that a conjugate of AgNPs, polyethylene glycol, and ciprofloxacin produced remarkably larger inhibition zones of 36 mm for *Acinetobacter baumannii*, 39 mm for *S. aureus*, and 40 mm for *Serratia marcescens* compared to the individual components. Also, Jalil et al.^79^ demonstrated that AgNPs conjugated with amoxicillin exhibited superior antimicrobial activity against *Streptococcus pneumoniae*, *S. aureus*, *P. aeruginosa*, and methicillin-resistant *S. aureus*, reinforcing the value of nanoparticle-mediated drug delivery systems.

The results of the MIC assays further substantiate the superior antibacterial efficacy of the Amp–AgNPs conjugate. The results highlight a synergistic interaction between ampicillin and AgNPs, enabling effective bacterial inhibition at significantly lower concentrations. Similarly, Khatoon et al.^74^ documented MICs of 18.75 µg/mL and 9.375 µg/mL for Amp–AgNPs against ampicillin-sensitive *E. coli* and *S. aureus*, respectively, and MICs of 10 µg/mL and 3 µg/mL for ampicillin-resistant counterparts. Also, Rogowska et al.^32^ demonstrated enhanced antibacterial efficacy using AgNPs functionalized with ampicillin, with MICs of 3.125 µg/mL for *P. aeruginosa*, 25 µg/mL for *E. coli*, and 6.25 µg/mL for *K. pneumoniae*. Antibiotic–AgNPs conjugates enhance antimicrobial efficacy with lower MICs, offering a more effective therapeutic strategy^36^. The effectiveness of the conjugation strategy is demonstrated by imipenem–AgNP conjugate, which exhibited markedly lower MICs (2–16 µg/mL) against 200 *P. aeruginosa* isolates compared to imipenem alone (64 to > 512 µg/mL) and AgNPs (4–32 µg/mL), highlighting their enhanced potency^88^. AgNPs functionalized with glucosamine demonstrated enhanced activity, with MICs as low as 8 µg/mL against methicillin-resistant *Staphylococcus aureus*^89^. These findings corroborate our current results and consistently support that conjugation of AgNPs with conventional antibiotics markedly reduces MIC values even in resistant strains.

The enhanced antibacterial efficacy of Amp–AgNPs conjugates can be attributed to multiple synergistic mechanisms involving the physicochemical properties of AgNPs and the biological activity of the antibiotics. Conjugation significantly enhances antibacterial activity against Gram-positive and Gram-negative bacteria, including MDR strains by enabling bioactive molecules to retain and even amplify their function when bound to the surface of AgNPs^90^. This enhanced potency likely results from the hydroxyl and amide groups of ampicillin and the AgNPs surface, which leads to improved cellular uptake and intracellular retention. Scanning electron microscopy analysis in the current study revealed pronounced morphological disruptions in *E. coli*, *E. faecalis*, and *S. aureus* treated with Amp–AgNPs, including membrane rupture, cytoplasmic leakage, and complete structural collapse, effects that were not observed with either ampicillin or AgNPs alone. AgNPs enhance antibiotic efficacy by increasing bacterial membrane permeability and facilitating greater intracellular drug uptake^35^. AgNPs disrupt bacterial membranes by binding to sulfur-containing proteins, compromising structural and enzymatic integrity, while also interfering with protein synthesis and DNA replication, ultimately causing irreversible cellular damage and death^70^. Conjugation enhances adhesion and membrane penetration through van der Waals and electrostatic interactions, disrupts DNA and metabolic processes, generates reactive oxygen species, and impairs electron transport, leading to bacterial cell death^91^. By integrating membrane disruption, intracellular interference, and enhanced delivery, the conjugates present a robust strategy for next-generation antimicrobial therapies, highlighting the need for further validation through in vivo studies.

### Effect of Amp–AgNPs conjugate on β-lactamases activity in clinical bacterial strains

**Fig. 8:**
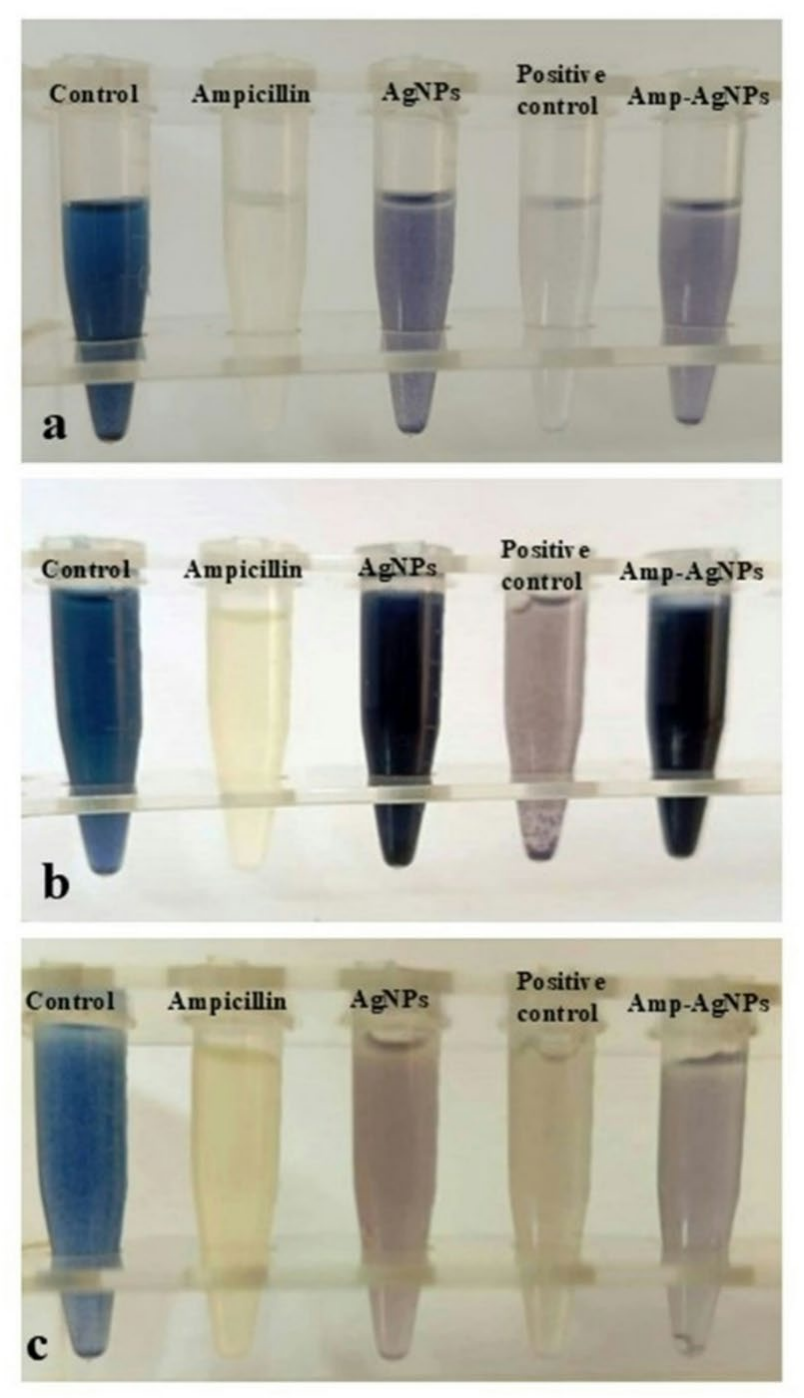
The effect of Amp–AgNPs on β-lactamase activity in bacterial strains assessed by iodometric assay: (**a**) *E. coli*, (**b**) *E. faecalis*, (**c**) *S. aureus*.

All tested strains (*E. coli*, *E. faecalis*, and *S. aureus*) were confirmed as β-lactamase producers, indicated by a distinct color change from blue to colorless in the iodometric assay, reflecting the enzymatic hydrolysis of free ampicillin (Fig. 8). The positive control (ampicillin/sulbactam) displayed a pale blue color, consistent with β-lactamase activity. In contrast, AgNPs and Amp–AgNPs maintained the blue color, demonstrating the absence of β-lactam ring hydrolysis. However, the slight color variation in the AgNPs and Amp–AgNPs samples is attributed to the adsorption of starch molecules onto the surfaces of the nanoparticles, rather than β-lactam ring hydrolysis, as AgNPs alone produced similar changes. All samples received identical volumes of iodine and starch, confirming that the observed differences arise from starch–nanoparticle interactions rather than reagent variation or enzymatic activity. This explanation is supported by reports showing that starch polymer networks act as active sites for nanoparticle adsorption^92^. The sustained blue coloration observed in the Amp–AgNPs treatment confirmed the stability of the conjugated form, suggesting that the β-lactam ring is shielded from enzymatic degradation.

The iodometric assay results revealed clear evidence of β-lactamase activity in *E. coli*, *E. faecalis*, and *S. aureus*, as indicated by the color change from blue to colorless upon ampicillin treatment. This confirms the susceptibility of conventional β-lactam antibiotics to enzymatic hydrolysis, a major mechanism underlying bacterial resistance^43,93^. In contrast, samples treated with Amp–AgNPs retained the blue coloration, indicating that this complex prevents β-lactamase activity and maintains ampicillin structural integrity. Amp–AgNPs demonstrated superior stability compared to the positive control, suggesting a protective role conferred by nanoparticle conjugation^94^.

A plausible explanation lies in the alteration of the steric and electronic characteristics of ampicillin upon conjugation with AgNPs. These modifications may prevent optimal substrate alignment within the β-lactamase active site, impairing catalytic function through steric hindrance or electronic redistribution^95^. This inhibition mechanism resembles competitive inhibition or substrate mimicry, a concept well-documented in the context of β-lactamase inhibitors^96^. Unlike classical inhibitors such as clavulanic acid, sulbactam, and tazobactam, which act through covalent acylation and irreversible enzyme inactivation^44^. This aligns with emerging efforts to develop non-β-lactam inhibitors capable of resisting enzymatic degradation across a broad spectrum of β-lactamase classes^37,97^. By preserving the β-lactam core and conferring enzymatic protection, Amp–AgNPs address the structural and functional limitations of existing β-lactamase inhibitors^40^. Due to the increasing prevalence of β-lactamase-mediated resistance and the decreasing effectiveness of current inhibitors^45^, hybrid nano-antibiotic platforms offer a promising new approach for antimicrobial drug design, providing renewed hope to combat resistance and enhance antibiotic effectiveness.

## Materials and methods

### Functional SDS-PAGE profiling and microplate assay confirmation of nitrate reductase involved in the biosynthesis and capping of AgNPs by *T. funiculosus*

The isolation and characterization of the proteins present in *T. funiculosus* filtrate and the corresponding biosynthesized AgNPs were conducted using SDS-PAGE as described by Laemmli^98^. Three distinct fungal filtrates were prepared from various patches of *T. funiculosus*, each utilized for synthesizing AgNPs as outlined by our previous study El deeb et al.^8^. The extracellular fungal filtrate of *T. funiculosus* was prepared by transferring 10 g (wet weight) of 4-day-old fungal mycelium into sterilized deionized water and incubating at 28 ± 2 °C for 72 h, followed by filtration to obtain a clear cell-free filtrate. The corresponding AgNPs were synthesized by reacting the filtrate with AgNO_3_ solution to a final concentration of 1 mM and incubating under dark conditions at 28 ± 2 °C for up to 72 h, during which a characteristic brown coloration developed, subsequently confirmed by UV–vis spectroscopy as evidence of AgNPs formation. The filtrates and the corresponding biosynthesized AgNPs were subsequently used to evaluate protein involvement in nanoparticle formation. The experiment was conducted in triplicate using independent fungal batches to ensure reproducibility and reliability of the results. SDS-PAGE analysis was performed on each fungal filtrate and its respective biosynthesized AgNPs to confirm protein presence and ensure the reproducibility of the results. Electrophoresis was conducted using a 12% SDS-polyacrylamide resolving gel within an Omni PAGE Mini Vertical Protein Electrophoresis System, UK, at a constant voltage of 100 V. After electrophoresis, the gel was stained with Coomassie Brilliant Blue dye and visualized using a gel imaging system (GelLITE, Cambridge, UK). Glyceraldehyde 3-phosphate dehydrogenase (GAPDH), with a molecular weight of 38 kDa, was run alongside the samples as an internal reference to validate the accuracy and reliability of molecular weight estimation. Also, a standard molecular weight marker (Promega, ADV8491, USA) was included to facilitate precise protein size determination.

To confirm the identity of the protein band detected by SDS-PAGE as nitrate reductase for the fungal filtrate and the biosynthesized AgNPs, a colorimetric microplate-based nitrate reductase assay was performed using a commercial kit (Nitrate Reductase Microplate Assay Kit, Cat. No. ORB219870, Biorbyt, USA). The assay quantified nitrate reductase activity based on the enzymatic reduction of nitrate to nitrite by chromogenic azo-compound detection under standard assay conditions. One unit of nitrate reductase activity was defined as the amount of enzyme required to generate 1 µmol of nitrite per hour. The assay was conducted in triplicate according to the manufacturer’s instructions, and enzyme activity was expressed as units per gram (U/g) of fungal biomass. Absorbance was measured at 540 nm using a Varioskan LUX multimode microplate reader (Thermo Fisher Scientific, Finland), and the data were analyzed using SkanIt Software for Microplate Readers RE, version 6.0.1.6. The detection of enzymatic activity corresponding to the 58 kDa protein band confirmed the functional presence of nitrate reductase in the fungal filtrate and the AgNPs, indicating its catalytic role in the biosynthesis and capping of AgNPs.

### Preparation and physicochemical characterization of Amp–AgNPs conjugate

Ampicillin solutions (0.2, 0.5, 1.0, 2.0, 3.0, and 4.0 mg/mL) were prepared in distilled water and mixed with biosynthesized AgNPs (1.0 mg/mL) to form Amp–AgNPs conjugate. Conversely, varying concentrations of AgNPs (0.2, 0.4, 0.6, 0.8, and 1.0 mg/mL) were mixed with ampicillin (1.0 mg/mL) to identify the optimal nanoparticle concentration for conjugation. The optimal concentration of ampicillin and AgNPs for functionalization was determined using UV–visible spectroscopy. The reaction mixtures were incubated at room temperature for 24 h and repeated in triplicate for reproducibility. The successful formation of the Amp–AgNPs conjugates was confirmed using multiple characterization techniques.

To monitor the surface Plasmon resonance (SPR) shifts, UV–visible spectra were conducted using a UV–visible spectrophotometer (JAS.CO V-770, Japan) within the wavelength range of 200–800 nm. Deionized water was used as a blank to adjust the baseline. Measurements were repeated after three months of preservation at room temperature to evaluate the long-term stability of the Amp–AgNPs.

The crystalline structure and phase purity of the Amp–AgNPs conjugate were analyzed by X-ray diffraction (XRD) in the 2θ range of 20°–90° using an X-ray diffractometer (D8 Advance, Germany) operated at 40 kV and 40 mA with Cu Kα radiation (λ = 1.54060 Å). Samples were prepared by drop-casting purified Amp–AgNPs suspensions onto glass substrates, followed by air-drying^99^.

The size distribution and average hydrodynamic diameter of Amp–AgNPs were assessed by DLS, while zeta potential (surface charge) was measured with a NANOTRAC WAVE II, Germany, to evaluate colloidal stability.

FTIR spectroscopy was conducted to explore the functional groups and potential interactions involved in the formation of the Amp–AgNPs conjugate. The spectra of pure ampicillin, biosynthesized AgNPs, and the conjugated complex were recorded using a Platinum-ATR FTIR spectrometer (Bruker Alpha, Germany) in the range of 4000–400 cm⁻¹. This spectral range facilitated the identification of characteristic functional groups and the assessment of possible chemical bonding or interactions between ampicillin and AgNPs.

Scanning electron microscopy (SEM) was performed using a Zeiss Sigma 500 VP field emission SEM (FESEM) to examine the morphological and surface characteristics of AgNPs before functionalization and the new Amp–AgNPs conjugate.

High-resolution transmission electron microscopy (HR-TEM) and selected area electron diffraction (SAED) analyses were performed to investigate the morphology, interactions, and crystalline structure of the Amp–AgNPs conjugate. These analyses were conducted using a JEOL JSM 100CX TEM instrument (JEOL Ltd., Japan) at the Electron Microscope Unit of Cairo University, Egypt. TEM imaging was used to evaluate the shape, size distribution, and dispersion of the nanoparticles, as well as to provide insight into the surface interaction and conjugation of ampicillin with AgNPs. The polydispersity index (PDI) was calculated from the measured particle sizes to assess the uniformity and dispersity of the nanoparticles. The PDI was obtained using Eq. (1): PDI = σ/R, where σ is the standard deviation of the particle size distribution obtained from TEM analysis and R is the mean particle diameter, as described by Rudrappa et al.^81^. SAED was utilized to confirm the crystalline nature of the nanoparticles and to evaluate any potential structural changes or aggregation that may have arisen during the conjugation process.

### Stability assessment of Amp–AgNPs under physiological pH and temperature conditions

The stability of the Amp–AgNPs conjugate was evaluated under varying pH and temperature conditions to assess its physicochemical robustness following the method described by Rogowska et al.^32^. For pH stability, aliquots were adjusted to pH values of 1, 3, 5, 7, 9, 11, and 13 using 0.1 M HCl or 0.1 M NaOH and incubated at room temperature for 48 h. Thermal stability was assessed by incubating separate aliquots at 5, 15, 25, 35, 45, and 55 °C for 48 h in a controlled water bath. UV–vis absorbance spectra (200–800 nm) were recorded to monitor changes in the characteristic peak at 340.5 nm, indicative of stable Amp–AgNPs conjugation. Shifts or reductions in peak intensity were used to evaluate conjugate integrity. The test was conducted in triplicate, and visual inspections were performed to detect signs of aggregation, turbidity, or precipitation, providing complementary evidence of colloidal stability.

### Computational investigation of ampicillin interaction with nitrate reductase enzyme capped AgNPs

Nitrate reductase enzyme’s sequence of *T. funiculosus* was obtained from the UniProt database (ID: F1CF56). The obtained sequence was used to search for a homology model using BLAST database, highly similar sequences with known crystal structures were used for multiple alignment and threading of the query enzyme (PDB IDs: 1SOX, 2A99, 2A9B, 2A9D, 2BIH, 2BII, 3HBG, 3HBP, 3HC2, 3R18, 3R19).

The 3D model of the enzyme was then designed using SWISSPdb^100^, where the template models were fitted together before threading the query sequence. This process enhances the modeling and decreases the clashes of the new model. The obtained model was validated using Ramachandran plot^101^. Furthermore, energy minimization was performed using GROMOS96 force field^102^. The obtained model was compared with the AI generator of Aphafold2, where the RMS value was found to be 1.55 Å. The obtained 3D model was used as a receptor for docking ampicillin substrate which was obtained from the PubChem database.

Docking protocols were created based on prior studies^103^ using USCF DOCK6^104^. The AM1-BBC method was used in DOCK6 to add charge to the protein and ampicillin. Utilizing a probe radius of 1 point 4 Å, the molecular surface was created. Using spheres within 9 Å of the native ligand, which is encircled by a box with a margin of 5 Å, the active site of a target was identified. Polar hydrogens were added to the targets in Vina, and then Kollman charges were added. In the meantime, ligand preparation involved the use of Gasteiger charges. To generate the center coordinate, a grid box was applied at a spacing of 1 Å, centered on the native ligand position. After comparing the ten conformations produced, docked structures were selected from the obtained conformations based on the binding energy (kcal/mol) of these conformations.

LIGPLOT^+^ (version 2.2) was used to visualize and determine the interactions between ligand and receptor’s amino acids. It examines the intricate receptor-ligand structure’s 2D hydrogen bond (HB) interaction. It provides a graphical representation of HB, hydrophobic bonds, and their bond lengths at the ideal docking position^105^.

### Integrated evaluation of antibacterial activity and morphological disruption by Amp–AgNPs conjugate against β-lactamase-producing bacteria

The antibacterial susceptibility test and the minimum inhibitory concentration (MIC) determinations were conducted as described by El deeb et al.^8^. The assays were performed against Gram-negative and Gram-positive β-lactamase-producing bacteria, including *Escherichia coli* (SUMCC 22014), *Enterococcus faecalis* (SUMCC 22015), and *Staphylococcus aureus* (SUMCC 22016). All strains were isolated from clinical samples obtained from the Sohag University Microbial Culture Collection, Egypt. Five concentrations (5, 10, 20, 30, 40, and 50 µg/mL) were tested for: ampicillin, biosynthesized AgNPs, positive control (ampicillin/sulbactam, 2:1 ratio), and the Amp–AgNPs conjugate. All experiments were performed in triplicate to ensure accuracy.

The antibacterial activity was evaluated using the disk diffusion method on Mueller–Hinton agar (MHA). Sterile filter paper disks were loaded with the designated concentrations of each tested compound. The selected concentration range for Amp–AgNPs was based on the typical daily intake of silver from natural sources, approximately 0.4–27 µg/day^106^, thus ensuring relevance to environmentally realistic exposure levels. Ampicillin served as the negative control to confirm bacterial resistance to ampicillin, while ampicillin/sulbactam was used as the positive control. The formed plates were incubated at 37 °C for 24 h, followed by measuring the formed inhibition zone in millimeters (mm).

The MIC for ampicillin, AgNPs, positive control (ampicillin/sulbactam), and Amp–AgNPs were determined for the tested pathogens by broth microdilution method using 96-well microtiter plates according to the principles described by Kowalska-Krochmal & Dudek-Wicher^107^. Concentrations ranging from 1 to 100 µg/mL, increasing by 1 µg/mL, were prepared to facilitate MIC determination. Resazurin, a blue dye that becomes pink upon reduction by metabolically active cells, was employed as an indicator of bacterial viability. The assay was performed in triplicate, and an inoculum was taken from each well that showed no visual growth and spotted on MHA plates to validate the MIC assay.

To evaluate the morphological alterations and potential molecular interactions induced by Amp–AgNPs, SEM analysis was performed on the tested bacterial cells before and after 6 h of exposure to the MIC of each tested compound. For ampicillin, a concentration of 100 µg/mL was used. The analysis included untreated control cells, cells treated with ampicillin, AgNPs, positive control (ampicillin/sulbactam), and the Amp–AgNPs conjugate. Morphological analysis was performed according to the protocol described by Singh et al.^108^ using a JEOL JSM-5400LV SEM (JEOL Ltd., Japan) operated at 15–25 kV.

### Impact of Amp–AgNPs conjugate on β-lactamases enzymatic activity

The iodometric method was employed to evaluate β-lactamase activity, following the protocols described by Sharma et al.^109^ and Aliyu et al.^110^. A loopful of a dense 24-hour culture of *E. coli*, *E. faecalis*, and *S. aureus* from MHA was mixed with 1.0 mL of a 10,000 µg/mL solution of ampicillin, AgNPs, ampicillin/sulbactam (β-lactamase inhibitor used as a positive control), and the Amp–AgNPs conjugate. The samples were incubated at room temperature for 30 min with gentle agitation at 15-minute intervals. After the incubation period, two drops of a 1% soluble starch solution and one drop of iodine solution were added to the mixture and gently shaken. A change in color from blue to colorless indicated positive β-lactamase activity, whereas the persistence of the blue color signified a negative result. Results were recorded within 10 min, and all tests were performed in triplicate for confirmation.

### Statistical analysis

All experiments were carried out in triplicate and the results were presented as mean ± standard deviation. The data were statistically analyzed by one-way analysis of variance (ANOVA) using XLSTAT version 2023.2.0 software^111^. Differences at *P* < 0.05 were regarded as statistically significant.

## Conclusion

This study successfully demonstrated the pivotal role of nitrate reductase (58 kDa) in the biosynthesis and stabilization of AgNPs by *Talaromyces funiculosus*. For the first time, the enzyme was shown to be retained and remains catalytically active on the nanoparticle surface, as evidenced by a 29.68% activity increase following AgNPs synthesis. Importantly, this is the first report of the biosynthesis of ampicillin–AgNPs conjugate mediated by nitrate reductase, where the characteristic SPR shift from 422.5 to 340.5 nm directly confirmed conjugation through strong drug-nanoparticle interactions. The conjugate was further validated by FTIR through the disappearance of the 1736 cm⁻¹ band and exhibited high physicochemical stability for 3 months, while still retaining its crystalline structure, with a semi-spherical morphology, polydisperse (PDI: 0.192), an average diameter of 27.26 nm, and a zeta potential of − 24.9 mV. Moreover, it demonstrated stability across a broad pH range (1–9) and temperature range (5–55 °C). In silico modelling of nitrate reductase revealed its active site architecture and demonstrated strong binding affinity with ampicillin via hydrogen bonding, hydrophobic, and electrostatic interactions, confirming conjugate stability. Functionally, Amp–AgNPs (50 µg/mL) significantly exceeded AgNPs and the positive control in inhibiting β-lactamase-producing bacterial strains and effectively restored the antimicrobial activity of ampicillin, with inhibition zones of 27.3 mm (*Escherichia coli*), 25.0 mm (*Enterococcus faecalis*), and 26.3 mm (*Staphylococcus aureus*), and remarkably low MICs of 3.3, 4.7, and 4.3 µg/mL, respectively. Moreover, Amp–AgNPs protected the antibiotic’s β-lactam ring from enzymatic degradation and caused pronounced bacterial ultra-structural damage, as confirmed by SEM analysis showing severe membrane disruption. Collectively, these findings establish a novel bio–nano interface strategy that utilizes nitrate reductase to construct stable antibiotic–nanoparticle conjugates with superior antimicrobial activity. The results highlight the potential of NR-mediated AgNPs as nano-carriers for antibiotic delivery, offering a promising route to overcome multidrug-resistant infections and advance antimicrobial nanobiotechnology. Future studies should explore in vivo efficacy, ensure biosafety, and extend this biomolecular strategy to a broader range of drug delivery applications.

## Supplementary Information

Below is the link to the electronic supplementary material.

Supplementary Material 1

## Data Availability

All data generated or analyzed during this study are included in this published article. Nitrate reductase enzyme’s sequence of *Talaromyces funiculosus* was obtained from the UniProt database (ID: F1CF56). The other datasets used and/or analyzed during the current study are available from the corresponding author upon request.
