# Supplementary material for: Biomolecular strategy for designing antibiotic–silver nanoparticles conjugate via nitrate reductase mediated β-lactamase inhibition with molecular docking insights
Source: Sci Rep. 2026 Jan 3;16:621. doi: 10.1038/s41598-025-30539-8 (PMC12775131; doi:10.1038/s41598-025-30539-8)
Supplement: Supplementary file 1 — Supplementary Material 1 [file 41598_2025_30539_MOESM1_ESM.pdf]

Biomolecular strategy for designing antibiotic–silver nanoparticles conjugate via nitrate reductase mediated β-lactamase inhibition with molecular docking insights

Gerges Gad Faheem[1], Bahig A. El Deeb[1,*], Mohamed Ismeal[2] & Mahmoud S. Bakhit[1]
[1]Department of Botany and Microbiology, Faculty of Science, Sohag University, Sohag, 82524, Egypt
[2]Department of Chemistry, Faculty of Science, Sohag University, Sohag, 82524, Egypt
*email: beldeep@science.sohag.edu.eg

Supplementary

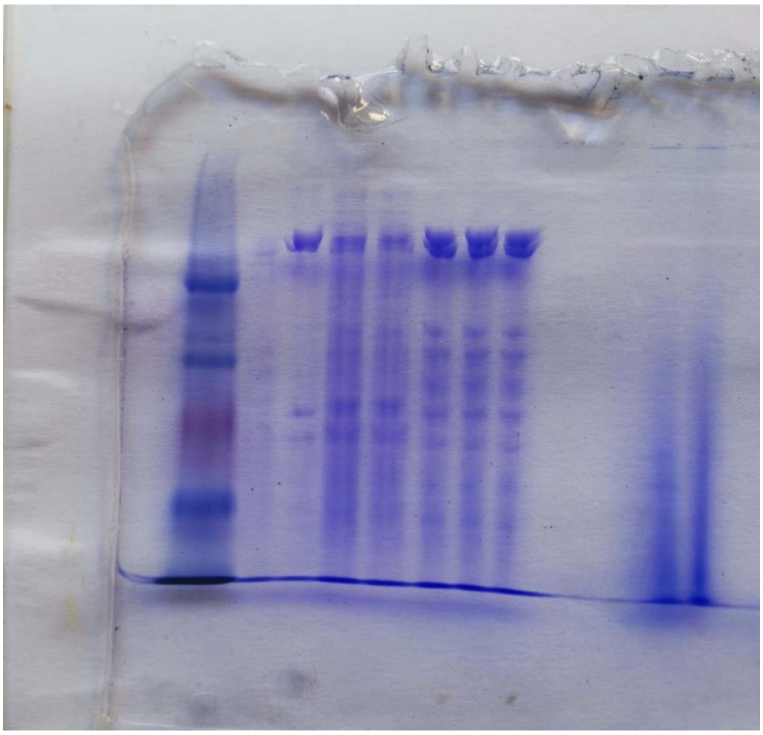

Supplemental Figure S1. Full SDS-PAGE gel image showing protein profiles in the *Talaromyces funiculosus* filtrate and the synthesized AgNPs.

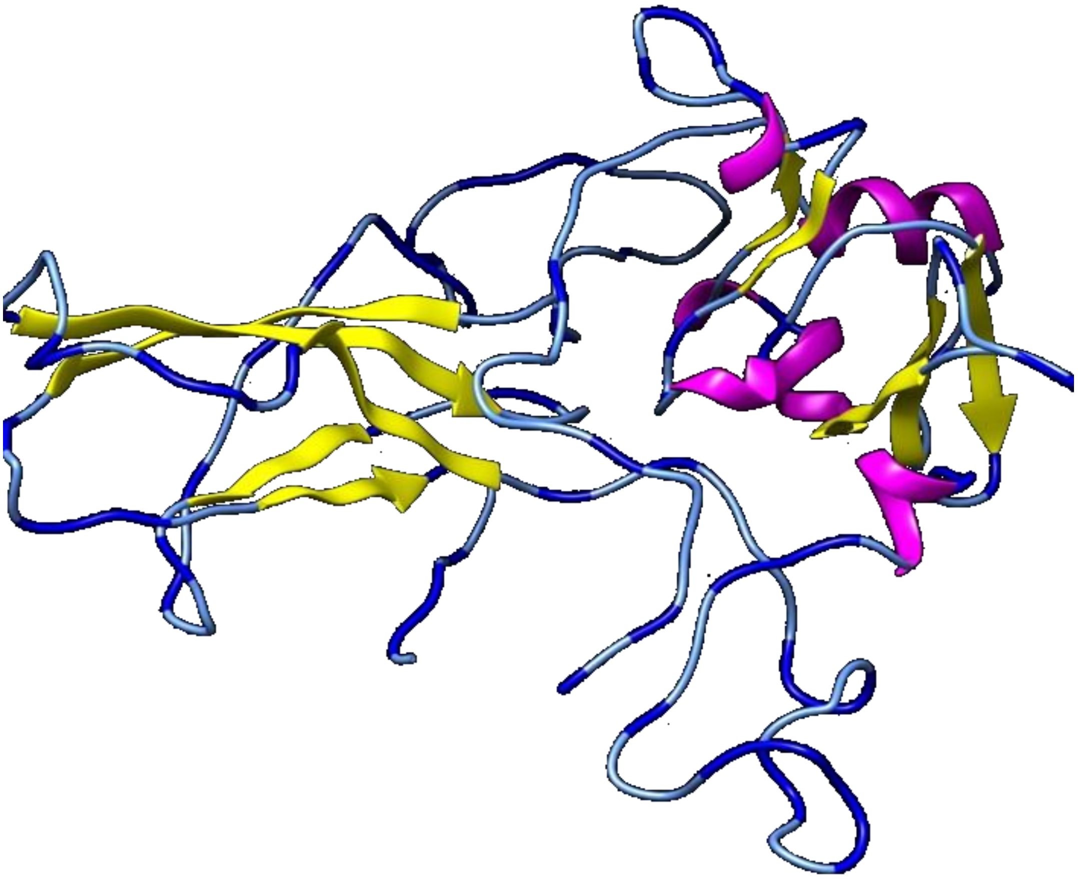

Supplemental Figure S2. 3D model of nitrate reductase built by homology modeling, helices are magenta, β-sheets are yellow, and coils are light and dark blue.

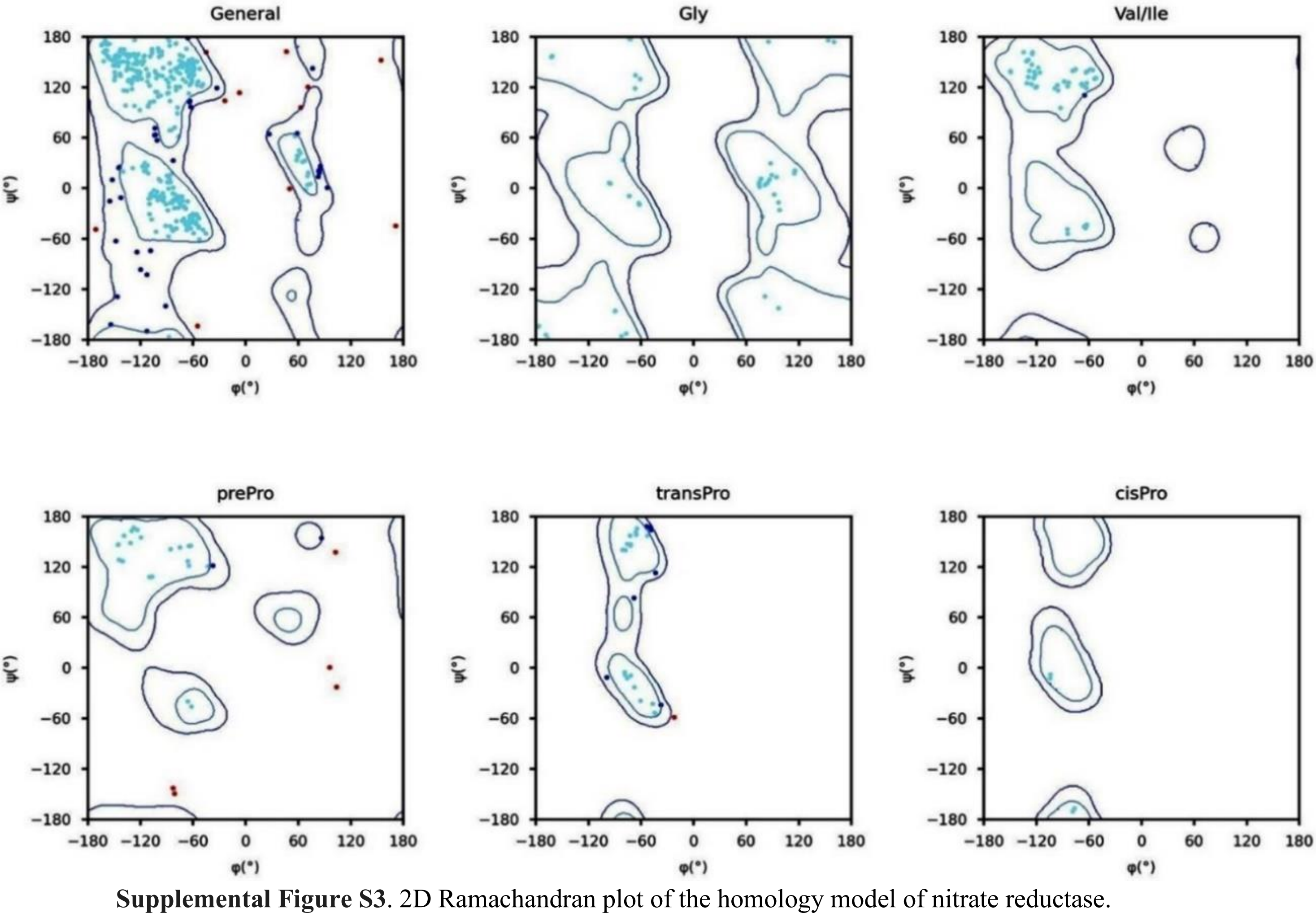

Supplemental Figure S3. 2D Ramachandran plot of the homology model of nitrate reductase.

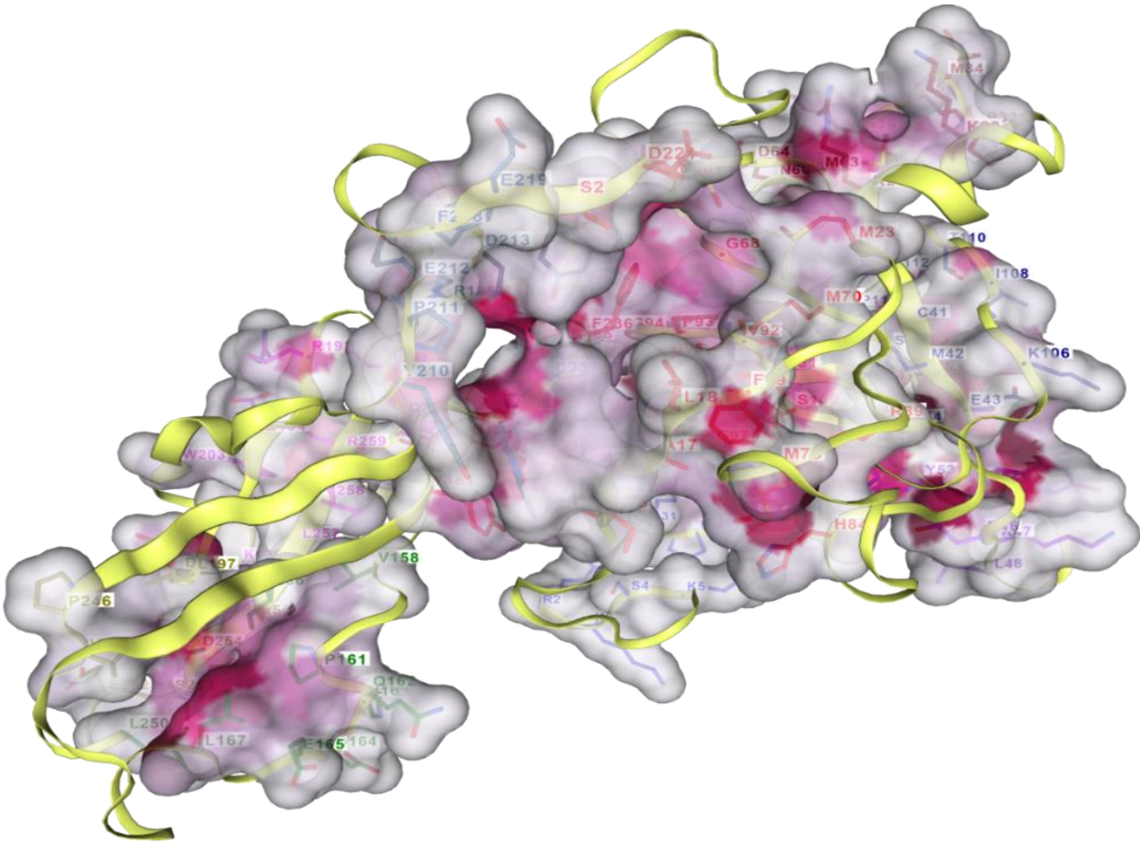

Supplemental Figure S4. Iso-surface representation of the pockets obtained by the cavity search tool.
